# Better ILP models for haplotype assembly

**DOI:** 10.1186/s12859-018-2012-x

**Published:** 2018-02-19

**Authors:** Maryam Etemadi, Mehri Bagherian, Zhi-Zhong Chen, Lusheng Wang

**Affiliations:** 1 Department of Applied Mathematics, Faculty of Mathematical Sciences, University of Guilan, Rasht, 41938-33697 Iran; 2 Division of Information System Design, Tokyo Denki University, Saitama, 350-0394 Japan; 3 Department of Computer Science, City University of Hong Kong, Kowloon, Hong Kong; 4 City University of Hong Kong Shenzhen Research Institute, ShenzhenHi-TechIndustrialPark, Nanshan District, Shenzhen, People’s Republic of China

**Keywords:** Haplotype assembly, ILP model, Minimun error correction, Diploid

## Abstract

**Background:**

The haplotype assembly problem for diploid is to find a pair of haplotypes from a given set of aligned Single Nucleotide Polymorphism (SNP) fragments (reads). It has many applications in association studies, drug design, and genetic research. Since this problem is computationally hard, both heuristic and exact algorithms have been designed for it. Although exact algorithms are much slower, they are still of great interest because they usually output significantly better solutions than heuristic algorithms in terms of popular measures such as the Minimum Error Correction (MEC) score, the number of switch errors, and the QAN50 score. Exact algorithms are also valuable because they can be used to witness how good a heuristic algorithm is. The best known exact algorithm is based on integer linear programming (ILP) and it is known that ILP can also be used to improve the output quality of *every heuristic algorithm* with a little decline in speed. Therefore, faster ILP models for the problem are highly demanded.

**Results:**

As in previous studies, we consider not only the general case of the problem but also its *all-heterozygous case* where we assume that if a column of the input read matrix contains at least one 0 and one 1, then it corresponds to a heterozygous SNP site. For both cases, we design new ILP models for the haplotype assembly problem which aim at minimizing the MEC score. The new models are theoretically better because they contain significantly fewer constraints. More importantly, our experimental results show that for both simulated and real datasets, the new model for the all-heterozygous (respectively, general) case can usually be solved via CPLEX (an ILP solver) **at least 5 times** (respectively, **twice**) **faster** than the previous bests. Indeed, the running time can sometimes be **41 times better**.

**Conclusions:**

This paper proposes a new ILP model for the haplotype assembly problem and its all-heterozygous case, respectively. Experiments with both real and simulated datasets show that the new models can be solved within much shorter time by CPLEX than the previous bests. We believe that the models can be used to improve heuristic algorithms as well.

## Background

Humans are diploid, i.e. their chromosomes except the sexual male ones consist of two copies, one inherited from the mother and the other inherited from the father. Most positions of a pair of chromosomes are formed of the same nucleotide. Based on International Hapmap Consortium 2007, the difference between two chromosomes of an individual or population is one among 1000 nucleotides [1–4]. If this difference is statistically meaningful (at least it is different in 5 percent of people) the site is called *single nucleotide polymorphism (SNP)*. Most of SNPs are bi-allelic, i.e. two of the four possible nucleotides appear in almost all the population. The alleles with high frequency are called *major* and coded into 0, and the alleles with low frequency are called *minor* and coded into 1 [5]. There are also sites whose nucleotides of the two copies of a chromosome are identical among all population. Such sites are ignored and SNPs are listed sequentially. The result is a binary string and is called the *haplotype*.

Haplotypes have many applications in prediction of diseases [6]; association studies [7–9], and drug design [10]. Unfortunately, the presence of sequencing errors makes it difficult and expensive to experimentally examine a single chromosome. This has motivated researchers to design algorithms for obtaining haplotypes from aligned reads. To deal with errors when looking for the best reconstruction of haplotypes, we have to fix an objective function for evaluating candidate haplotypes and solve an optimization problem (called the *haplotype assembly problem*). Many different objective functions have been proposed, including Minimum Fragment Removal (MFR), Minimum SNP Removal (MSR), Longest Haplotype Reconstruction (LHR), Minimum Implicit SNP Removal (MISR), Minimum Implicit Fragment Removal (MIFR), MEC [11, 12], and Maximum Fragments Cut (MFC) [13]. Of special interest among the proposed functions is MEC, which aims at minimizing the total number of conflicts (errors) between the reads and the constructed haplotypes. MFC is another interesting function. However, Chen et al. [14] found out that optimizing MEC leads to better output haplotypes than MFC (in terms of popular measures such as the number of switch errors and the QAN50 score).

As one can expect, the problem of minimizing MEC is NP-hard even for gapless reads [12, 15]. This has motivated researchers to design both heuristic and exact algorithms for the problem. Wang et al. [16] designed a branch-and-bound algorithm to solve the problem to optimality. But due to the exponential complexity of their algorithm, they proposed a genetic algorithm for large scale problems. Using Sanger sequencing technology, Levy et al. [17] introduced the first diploid genome sequence of an individual human, referred as *HuRef*, and proposed a greedy heuristic method for concatenating the reads with minimum conflicts. Although their method is fast, it becomes inaccurate when errors are present in reads. A heuristic method (called *Fast Hare*) was proposed in [18]. He et al. [19] introduced a dynamic-programming algorithm with the complexity of *O*(2^*k*^*mn*), where *k* is the length of the longest read, *m* is the number of reads, and *n* is the total number of SNPs in the haplotypes. Their algorithm works well when *k*≤15. However, when *k* is large, they have to model the problem as a MaxSAT problem, which should be solved by a MaxSAT solver. In comparison to dynamic programming, Fast Hare is much faster and more accurate. The complexity of Fast Hare is *O*(*n* log*n*+*mn*), where *n* and *m* are the number of fragments and SNPs, respectively. Bansal et al. [20] introduced a software package (named *HapCUT*), in which the algorithm tries to minimize the MEC score by iteratively computing max-cuts in graphs derived from the sequenced fragments. Bansal et al. [21] developed a Markov chain Monte Carlo algorithm (called *HASH*). Both HASH and HapCut work well in practice, but may not find optimal haplotypes. Mousavi et al. [22] developed an improved maximum likelihood model which solves the problem approximately. They also showed that the maximum likelihood model is approximated by the MEC model when the minor allele frequencies are ignored from the model and in this way provides a theoretical support for the MEC model despite some criticisms against it [13]. Bonizzoni et al. [23] provided new results on the fixed-parameter tractability and approximability of MEC and showed that MEC is fixed-parameter tractable (FPT) when parameterized by the number of corrections; moreover, on “gapless” instances, it is also FPT when parameterized by the length of the fragments. Ahn et al. [24] developed a probabilistic framework and a sequential Monte Carlo algorithm (called *ParticleHap*), which employs a deterministic sequential Monte Carlo algorithm that associates SNPs with haplotypes by exhaustively exploring all possible extensions of the partial haplotypes. Das et al. [25] formulated the problem as a semi-definite program and using the low rank of the underlying solution, solved it fast and with high accuracy. Rhee et al. [26] surveyed the development of computational haplotype determination methods.

Chen et al. [27] introduced a novel approach via which we can solve the problem to optimality. Their algorithm proceeds as follows. First, we reduce the size of the input read matrix and further partition the problem into a number of independent smaller problems whose optimal solutions can be combined into an optimal solution of the original problem. To solve each smaller problem, they formulate it as an ILP problem and use a fast ILP solver (such as CPLEX and GUROBI) to solve it. With their approach, they succeed in finding optimal haplotypes for the difficult HuRef dataset on a single PC within 31 h in total.

When solving the haplotype assembly problem, we usually make the *all-heterozygous assumption* which assumes that if a column of the input read matrix contains at least one 0 and one 1, then it corresponds to a heterozygous SNP site. It is well known that the all-heterozygous assumption is correct for most columns in the read matrix but it may be incorrect due to errors in reads. So, it is natural to consider the *general case* of the problem, where we do not make the assumption. The general case is much harder than the all-heterozygous case, because it allows many more candidate haplotypes. Nonetheless, Chen et al. [27] show that their ILP-based approach to the all-heterozygous case can be generalized to solve the general case as well. In particular, they show that even in the general case, we can solve the HuRef dataset (except a single subproblem for the 15th chromosome) on a single PC within a total of 12 days. For convenience, we refer to their model for the all-heterozygous (respectively, general) as *OldModelA* (respectively, *OldModelG*).

To the best of our knowledge, Chen et al.’s approach [27] leads to the previously fastest algorithm for optimally solving the problem. However, their program can take hours or even days for difficult instances. Hence, it is of great interest if we can design better ILP models that lead to significant speed-up of Chen et al.’s approach. In this paper, we come up with such models for both the all-heterozygous and the general cases. The new models are theoretically better because they contain significantly fewer constraints. More importantly, our experimental results show that for both simulated and real datasets, the new model for the all-heterozygous (respectively, general) case can usually be solved via CPLEX (a popular ILP solver) **at least 5 times** (respectively, **twice**) **faster** than the previously best models. Indeed, the running time can sometimes be **41 times better**. The real datasets we use are HuRef [17] and MP1 [28], while the simulated dataset is Geraci’s dataset [29] and Chen et al.’s Simu95_MP1 [14].

One may argue that exact algorithms are much slower than heuristic algorithms for difficult instances. However, exact algorithms are still of great interest for the following reasons. First, as found out in [14], exact algorithms usually output significantly better solutions than heuristic algorithms in terms of popular measures such as the MEC score, the number of switch errors, and the QAN50 score. Secondly, exact algorithms can be used to witness how good a heuristic algorithm is. Thirdly, as found out in [14], ILP models of the problem can be used to improve the output quality of *every heuristic algorithm* with a little decline in speed. Thus, better ILP models also lead to faster improvement of heuristic algorithms.

## Methods

One method for obtaining haplotype information is to collect genotype data from an individual. Genotype data indicate information of the set of alleles at each locus, but it is not known on which chromosome a particular allele occurs [30–32]. Other methods are based on the data of shotgun sequence assembly [12, 21, 33]. Given a reference genome sequence and a set of reads containing sequences of both chromosomes, first of all we need to align the reads to the reference genome. We keep only those columns with multiple (different) values in the alignment. These columns are called the *heterozygous sites* and correspond to alleles that differ between chromosomes. For each heterozygous site, the major and minor alleles are coded into 0 and 1, respectively. In this way, we obtain aligned SNP fragments as the input data. Then, we need to partition the aligned SNP fragments into two sets according to certain objective function and further determine a haplotype from each set via the majority rule. Formally, the input data is a matrix *A* whose rows and columns correspond to reads and SNP sites, respectively. Each entry of *A* is a 0, 1, or ‘ −’, where ‘ −’ is called a *gap* and either corresponds to missing data or serves as gaps to connect disjoint parts of the read. The first (respectively, last) entry of a read that is not a ‘ −’ is called the *start* (respectively, *end*) position of the read. Note that there may exist gaps between the start and the end positions of a read. The following is an example read matrix:

$$A=\left[ \begin{array}{cccccc} - & 1 & 0 & 0 & - & - \\ 1 & 1 & - & 0 & - & - \\ 0 & 1 & - & 1 & 0 & 1 \\ - & 0 & 1 & - & - & - \\ 1 & - & - & 0 & 1 & - \\ 1 & 0 & 1 & - & - & 0 \\ \end{array} \right] $$

Each row of *A* can be viewed as a ternary string of length *m* over {0,1,−}, where *m* is the number of columns in *A*. For two ternary strings *s* and *t* of the same length, we use *d*(*s,t*) to denote the total number of positions at which both characters of *s* and *t* belong to {0,1} but they differ. Given *A*, the haplotype assembly problem requires the computation of a pair (*h,h*^′^) of binary strings with length *m* whose *MEC score*, i.e., $\sum _{r}\min \{d(h,r), d(h',r)\}$ is minimized, where *r* ranges over all rows of *A*. For convenience, we call (*h,h*^′^) an *optimal pair* of haplotypes, and say that *r* is aligned to *h* (respectively, *h*^′^) if *d*(*h,r*)≤*d*(*h*^′^,*r*) (respectively, *d*(*h*^′^,*r*)<*d*(*h,r*)).

In the all-heterozygous case of the problem, it is required that *h* and *h*^′^ are *complementary*, i.e., each letter of *h* differs from the letter of *h*^′^ at the same position, while in the general case, a letter of *h* can equal the letter of *h*^′^ at the same position. The general case is more difficult to solve than the all-heterozygous case because it allows many more candidate haplotypes. To make the general case less difficult, Chen et al. [27] prove a lemma which can be used to find *intrinsically heterozygous columns*, i.e., those columns in *A* at which the letters of *h* and *h*^′^ can be assumed to differ without altering the optimal MEC score. Of course, if a column in *A* is not known to be intrinsically heterozygous, then the column may be heterozygous or homozygous, i.e., the letters of *h* and *h*^′^ at the column may differ or equal.

### Reductions

Suppose that *A* is the given read matrix. Clearly, we can assume that each row of *A* contains at least one 0 or 1 because otherwise the row can be deleted without changing the problem. We want to reduce the problem for *A* to smaller problems whose optimal solutions can be combined into an optimal solution of the original problem. The following three reductions are obvious and have been used before [27]. The first reduction removes all *monotone columns*, i.e., those columns in which at least one of 0 and 1 does not appear. Note that if one does not want such a column to be deleted by our program because the column is known to be heterozygous, then he/she should simply add a new read which starts and ends at the column so that both 0 and 1 appear in the column. The second reduction is called *Singleton-Removal*; it removes all rows in which only one column is not a ‘ −’ and the column is known to be (intrinsically) heterozygous. Let *m* be the number of columns in *A*. The third reduction first finds the set *S* of all *i*∈{1,…,*m*−1} such that for each read *r* in *A*, either the end position of *r* is in the *j*-th column for some *j*≤*i* or the start position of *r* is in the *k*-th column for some *k*≥*i*+1; and then partition *A* into |*S*|+1 (smaller) matrices by cutting *A* vertically at the border between the *i*-th and the (*i*+1)st columns for each *i*∈*S*. Each of the |*S*|+1 matrices is called a *block*.

Consider a block *B* of *A*. Let $\hat {m}$ be the number of columns in *B*. Chen et al. [27] suggests a less obvious reduction which can be used to reduce the problem for *B* to even smaller problems whose optimal solutions can be combined into an optimal solution of the problem for *B*. More specifically, the reduction first finds the set *T* of all $i\in \{2,\ldots,\hat {m}-1\}$ such that (1) the *i*-th column of *B* is known to be (intrinsically) heterozygous and (2) for each read *r* in *B*, either the end position of *r* is in the *j*-th column with *j*≤*i* or the start position of *r* is in the *k*-th column with *k*≥*i*. The reduction then modifies *B* by making a copy of the *i*-th column and putting it immediately after the original *i*-th column for all *i*∈*T*. In this way, the modified *B* has |*T*| more columns than the original *B*. Finally, the reduction splits the modified *B* into |*T*|+1 (smaller) matrices by cutting the modified *B* vertically at the border between the original *i*-th column and its duplicate for each *i*∈*T*. Each of the |*T*|+1 matrices is called a *reduced block*. If *T*≠*∅*, then the Singleton-Removal reduction can be applied to each reduced block $\tilde {B}$ because at least one read in $\tilde {B}$ starts and ends at the same (intrinsically) heterozygous column which is the first or the last column of $\tilde {B}$.

### Solving reduced blocks via ILP

Let $\tilde {B}$ be a reduced block. We want to formulate the haplotype assembly problem for $\tilde {B}$ as an ILP problem in both the general and the all-heterozygous cases. Towards this end, we first need a definition. Two columns *c* and *c*^′^ of $\tilde {B}$ are *complementary* if *c* can be obtained from *c*^′^ by flipping each 0 (respectively, 1) in *c*^′^ to a 1 (respectively, 0). The point is that if *c* and *c*^′^ are complementary, then there must exist an optimal pair (*h,h*^′^) of haplotypes for $\tilde {B}$ such that the letters of *h* (respectively, *h*^′^) at *c* and *c*^′^ are complementary and hence it suffices to compute the letter of *h* (respectively, *h*^′^) at *c* instead of computing the letters of *h* (respectively, *h*^′^) at both *c* and *c*^′^. Clearly, similar observations hold for identical rows and columns in $\tilde {B}$. Based on these observations, we first partition the rows (respectively, columns) of $\tilde {B}$ into disjoint sets so that the rows (respectively, columns) in the same set are identical (respectively, identical or complementary) but no two rows (respectively, columns) in different sets are, and then for each set *S* in the partition, modify $\tilde {B}$ by merging the rows (respectively, columns) in *S* into a single row (respectively, column) to which we assign the multiplicity |*S*|.

Suppose that we have modified $\tilde {B}$ as above. Let *p* (respectively, *q*) be the number of rows (respectively, columns) in $\tilde {B}$. For each *i*∈{1,…,*p*}, let *w*_*i*_ be the multiplicity of the *i*-th row of $\tilde {B}$. Similarly, for each *j*∈{1,…,*q*}, let *c*_*j*_ be the multiplicity of the *j*-th column of $\tilde {B}$.

#### The all-heterozygous case

For each integer *i* with 1≤*i*≤*p*, let *J*_*i*,0_ (respectively, *J*_*i*,1_) be the set of integers *j*∈{1,2,…,*q*} such that the *i*-th entry in the *j*-th column of $\tilde {B}$ is a 0 (respectively, 1). Since we want to compute an optimal pair (*h,h*^′^) of complementary haplotypes for $\tilde {B}$, we introduce a binary variable *x*_*j*_ for the *j*-th column whose value is supposed to be 1 if and only if the *j*-th bit of *h* is a 1 (and hence the *j*-th bit of *h*^′^ is a 0). Moreover, we introduce a binary variable *z*_*i*_ for the *i*-th row of $\tilde {B}$ whose value is supposed to be 1 if and only if the *i*-th row of $\tilde {B}$ is aligned to *h*. Furthermore, we introduce a binary variable *t*_*i,j*_ for the *i*-th row and the *j*-th column of $\tilde {B}$ whose value is supposed to be 1 if and only if the *i*-th row of $\tilde {B}$ is aligned to *h* but its *j*-th entry is different from that of *h*. Then, we claim that the problem of finding an optimal pair (*h,h*^′^) of complementary haplotypes for $\tilde {B}$ has the following ILP formulation (denoted by NewModelA):

$$\begin{array}{@{}rcl@{}} \text{Minimize} & &\sum_{i=1}^{p} w_{i} \sum_{j \in J_{i,0}} c_{j} \left(1 - x_{j} - z_{i} + 2 t_{i,j}\right) \\ &+& \sum_{i=1}^{p} w_{i} \sum_{j \in J_{i,1}} c_{j} \left(x_{j} - z_{i} + 2 t_{i,j}\right) \\ \text{Subject to} & & \\ & &\forall_{1\le i\le p} \ \ \forall_{j\in J_{i,0}} \ \ x_{j} + z_{i} - 1 \le t_{i,j} \\ & &\forall_{1\le i\le p} \ \ \forall_{j\in J_{i,1}} \ \ z_{i} - x_{j} \le t_{i,j} \\ & & \text{all the variables \(x_{j}\), \(z_{i}\), \(t_{i,j}\)~:~binary} \end{array} $$

To prove the above claim, we first show that for each *i*∈{1,…,*p*} and each *j*∈*J*_*i*,0_, (1−*x*_*j*_−*z*_*i*_+2*t*_*i,j*_)=1 if and only if either *z*_*i*_=1 and *x*_*j*_=1, or *z*_*i*_=0 and *x*_*j*_=0. Note that *z*_*i*_=1 and *x*_*j*_=1 together mean that the *i*-th row is aligned to *h* but its *j*-th entry is different from that of *h*, while *z*_*i*_=0 and *x*_*j*_=0 together mean that the *i*-th row is aligned to *h*^′^ but its *j*-th entry is different from that of *h*^′^. In Table 1, we enumerate the four possible values of (*x*_*j*_,*z*_*i*_). As can be seen from the table, *t*_*i,j*_ also happens to be the minimum binary variable to bound *x*_*j*_+*z*_*i*_−1 from above. Now, since the coefficient of *t*_*i,j*_ in the objective function is positive, minimizing the objective function leads to minimizing *t*_*i,j*_ and hence the condition *x*_*j*_+*z*_*i*_−1≤*t*_*i,j*_ (together with the fact that *t*_*i,j*_ is binary) guarantees that *t*_*i,j*_ is indeed as defined.

**Table 1:** The value of (1−*x*_*j*_−*z*_*i*_+2*t*_*i,j*_) for *j*∈*J*_*i*,0_

*x*_*j*_	*z*_*i*_	*x*_*j*_+*z*_*i*_−1	*t*_*i,j*_	(1−*x*_*j*_−*z*_*i*_+2*t*_*i,j*_)
0	0	− 1	0	1
0	1	0	0	0
1	0	0	0	0
1	1	1	1	1

To finish the proof of the claim, it suffices to show that for each *i*∈{1,…,*p*} and each *j*∈*J*_*i*,1_, (*x*_*j*_−*z*_*i*_+2*t*_*i,j*_)=1 if and only if either *z*_*i*_=1 and *x*_*j*_=0, or *z*_*i*_=0 and *x*_*j*_=1. The proof is similar to the case where *j*∈*J*_*i*,0_; the main difference is to use Table 2.

**Table 2:** The value of (*x*_*j*_−*z*_*i*_+2*t*_*i,j*_) for *j*∈*J*_*i*,1_

*x*_*j*_	*z*_*i*_	*z*_*i*_−*x*_*j*_	*t*_*i,j*_	(*x*_*j*_−*z*_*i*_+2*t*_*i,j*_)
0	0	0	0	0
0	1	1	1	1
1	0	− 1	0	1
1	1	0	0	0

We next compare NewModelA against Chen et al.’s OldModelA [27] in terms of the numbers of variables and constraints. OldModelA is as follows:

$$\begin{array}{@{}rcl@{}} \text{Minimize} & &\sum_{i=1}^{p} w_{i} \sum_{j \in J_{i,0}} c_{j} \left(1 - x_{j} - z_{i} + 2 t_{i,j}\right) \\ &+& \sum_{i=1}^{p} w_{i} \sum_{j \in J_{i,1}} c_{j} \left(z_{i} + x_{j} - 2 t_{i,j}\right) \\ \text{Subject to} & & \\ & & \forall_{1\le i\le p} \ \ \forall_{j\in J_{i,0} \cup J_{i,1}} \ \ t_{i,j} \le z_{i} \\ & & \forall_{1\le i\le p} \ \ \forall_{j\in J_{i,0} \cup J_{i,1}} \ \ t_{i,j} \le x_{j} \\ & & \forall_{1\le i\le p} \ \ \forall_{j\in J_{i,0} \cup J_{i,1}} \ \ t_{i,j} \ge z_{i} + x_{j} -1 \\ & & \text{all the variables \(x_{j}\), \(z_{i}\), \(t_{i,j}\)~:~binary} \end{array} $$

The number of variables in NewModelA is *p*+*q*+*pq* and so is that in OldModelA. On the other hand, the number of constraints in NewModelA is $\sum _{i=1}^{p} (|J_{i,0}| + |J_{i,1}|)$, while that in OldModelA is $3\sum _{i=1}^{p} (|J_{i,0}| + |J_{i,1}|)$. So, NewModelA has significantly fewer constraints. Thus, it looks very promising that NewModelA can almost always be solved in much shorter time. In a later section, we will see that this is actually the case.

#### The general case

For each integer *i* with 1≤*i*≤*p*, let *J*_*i*,0_ (respectively, *J*_*i*,1_) be the set of integers *j*∈{1,2,…,*q*} such that the *j*-th column of $\tilde {B}$ is known to be intrinsically heterozygous and the *i*-th entry in the *j*-th column of $\tilde {B}$ is a 0 (respectively, 1). Further let $\overline {J_{i,0}}$ (respectively, $\overline {J_{i,1}}$) be the set of integers *j*∈{1,2,…,*q*} such that the *j*-th column of $\tilde {B}$ is not known to be intrinsically heterozygous and the *i*-th entry in the *j*-th column of $\tilde {B}$ is a 0 (respectively, 1). As in the all-heterozygous case, we introduce a binary variable *z*_*i*_ for the *i*-th row of $\tilde {B}$ whose value is supposed to be 1 if and only if the *i*-th row of $\tilde {B}$ is aligned to *h*. For each *j*∈{1,…,*q*} such that the *j*-th column of $\tilde {B}$ is known to be intrinsically heterozygous, we introduce a binary variable *x*_*j*_ whose value is supposed to be 1 if and only if the *j*-th bit of *h* is a 1 (and hence the *j*-th bit of *h*^′^ is a 0). Moreover, for each *j*∈{1,…,*q*} such that the *j*-th column of $\tilde {B}$ is not known to be intrinsically heterozygous, we introduce two binary variables *x*_*j*_ and *y*_*j*_, where the value of *x*_*j*_ (respectively, *y*_*j*_) is supposed to be 1 if and only if the *j*-th bit of *h* (respectively, *h*^′^) is a 1. Furthermore, we introduce a binary variable *t*_*i,j*_ for the *i*-th row and the *j*-th column of $\tilde {B}$ whose value is supposed to be 1 if and only if the *i*-th row of $\tilde {B}$ is aligned to *h* but its *j*-th entry is different from that of *h*. In addition, if the *j*-th column of $\tilde {B}$ is not known to be intrinsically heterozygous, we further introduce a binary variable *u*_*i,j*_ whose value is supposed to be 1 if and only if the *i*-th row of $\tilde {B}$ is aligned to *h*^′^ but its *j*-th entry is different from that of *h*^′^.

Then, we claim that the problem of finding an optimal pair (*h,h*^′^) of haplotypes for $\tilde {B}$ has the following ILP formulation (denoted by NewModelG):

$$\begin{array}{@{}rcl@{}} \text{Minimize} & &\sum_{i=1}^{p} w_{i} \sum_{j \in J_{i,0}} c_{j} \left(1 - x_{j} - z_{i} + 2 t_{i,j}\right) \\ &+& \sum_{i=1}^{p} w_{i} \sum_{j \in J_{i,1}} c_{j} \left(x_{j} - z_{i} + 2 t_{i,j}\right) \\ &+& \sum_{i=1}^{p} w_{i} \cdot \sum_{j \in \overline{J_{i,0}}\cup \overline{J_{i,1}}} c_{j} \left(t_{i,j} + u_{i,j}\right) \\ \text{Subject to} & & \\ & &\forall_{1\le i\le p} \ \ \forall_{j\in J_{i,0}\cup\overline{J_{i,0}}} \ \ x_{j} + z_{i} - 1 \le t_{i,j} \\ & &\forall_{1\le i\le p} \ \ \forall_{j\in J_{i,1}\cup\overline{J_{i,1}}} \ \ z_{i} - x_{j} \le t_{i,j} \\ & &\forall_{1\le i\le p} \ \ \forall_{j\in \overline{J_{i,0}}} \ \ y_{j} - z_{i} \le u_{i,j} \\ & &\forall_{1\le i\le p} \ \ \forall_{j\in \overline{J_{i,1}}} \ \ 1 - y_{j} - z_{i} \le u_{i,j} \\ & & \text{all the variables \(x_{j}\), \(y_{j}\), \(z_{i}\), \(t_{i,j}\), \(u_{i,j}\)~:~binary} \end{array} $$

To prove the above claim, we first show that for each *i*∈{1,…,*p*} and each $j\in \overline {J_{i,0}}$, *t*_*i,j*_=1 if and only if *z*_*i*_=1 and *x*_*j*_=1, while *u*_*i,j*_=1 if and only if *z*_*i*_=0 and *y*_*j*_=1. Note that *z*_*i*_=1 and *x*_*j*_=1 together mean that the *i*-th row is aligned to *h* but its *j*-th entry is different from that of *h*, while *z*_*i*_=0 and *y*_*j*_=1 together mean that the *i*-th row is aligned to *h*^′^ but its *j*-th entry is different from that of *h*^′^. In Table 3, we enumerate the eight possible values of (*x*_*j*_,*y*_*j*_,*z*_*i*_). As can be seen from the table, *t*_*i,j*_ (respectively, *u*_*i,j*_) also happens to be the minimum binary variable to bound *x*_*j*_+*z*_*i*_−1 (respectively, *y*_*j*_−*z*_*i*_) from above. Now, since the coefficients of *t*_*i,j*_ and *u*_*i,j*_ in the objective function are positive, minimizing the objective function leads to minimizing *t*_*i,j*_ and *u*_*i,j*_ and hence the condition *x*_*j*_+*z*_*i*_−1≤*t*_*i,j*_ (respectively, *y*_*j*_−*z*_*i*_≤*u*_*i,j*_) together with the fact that *t*_*i,j*_ (respectively, *u*_*i,j*_) is binary guarantees that *t*_*i,j*_ (respectively, *u*_*i,j*_) is indeed as defined.

**Table 3:** The value of (*t*_*i,j*_+*u*_*i,j*_) for $j \in \overline {J_{i,0}}$

*x*_*j*_	*y*_*j*_	*z*_*i*_	*x*_*j*_+*z*_*i*_−1	*y*_*j*_−*z*_*i*_	*t*_*i,j*_	*u*_*i,j*_	(*t*_*i,j*_+*u*_*i,j*_)
0	0	0	− 1	0	0	0	0
1	0	0	0	0	0	0	0
0	1	0	− 1	1	0	1	1
1	1	0	0	1	0	1	1
0	0	1	0	− 1	0	0	0
0	1	1	0	0	0	0	0
1	0	1	1	− 1	1	0	1
1	1	1	1	0	1	0	1

Continuing the proof of the claim, we next show that for each *i*∈{1,…,*p*} and each $j\in \overline {J_{i,1}}$, *t*_*i,j*_=1 if and only if *z*_*i*_=1 and *x*_*j*_=0, while *u*_*i,j*_=1 if and only if *z*_*i*_=0 and *y*_*j*_=0. The proof is similar to the case where $j\in \overline {J_{i,0}}$; the main difference is to use Table 4.

**Table 4:** The value of (*t*_*i,j*_+*u*_*i,j*_) for $j \in \overline {J_{i,1}}$

*x*_*j*_	*y*_*j*_	*z*_*i*_	*z*_*i*_−*x*_*j*_	1−*y*_*j*_−*z*_*i*_	*t*_*i,j*_	*u*_*i,j*_	(*t*_*i,j*_+*u*_*i,j*_)
0	0	0	0	1	0	1	1
1	0	0	− 1	1	0	1	1
0	1	0	0	0	0	0	0
1	1	0	− 1	0	0	0	0
0	0	1	1	0	1	0	1
0	1	1	1	− 1	1	0	1
1	0	1	0	0	0	0	0
1	1	1	0	− 1	0	0	0

Now, by the analysis in the all-heterozygous case, the claim holds.

We next compare NewModelG against Chen et al.’s OldModelG [27] in terms of the numbers of variables and constraints. OldModelG is as follows:

$$\begin{array}{@{}rcl@{}} \text{Minimize} & &\sum_{i=1}^{p} w_{i} \sum_{j \in J_{i,0}} c_{j} \left(1 - x_{j} - z_{i} + 2 t_{i,j}\right) \\ &+& \sum_{i=1}^{p} w_{i} \sum_{j \in J_{i,1}} c_{j} \left(z_{i} + x_{j} - 2 t_{i,j}\right) \\ &+& \sum_{i=1}^{p} w_{i} \cdot \sum_{j \in \overline{J_{i,0}}} c_{j} \left(y_{j} + t_{i,j} - u_{i,j} \right) \\ &+& \sum_{i=1}^{p} w_{i} \cdot \sum_{j \in \overline{J_{i,1}}} c_{j} \left(1 - y_{j} - t_{i,j} +u_{i,j}\right) \\ \text{Subject to} & & \\ & &\forall_{1\le i\le p} \ \ \forall_{j\in J_{i,0}\cup J_{i,1}\cup\overline{J_{i,0}}\cup\overline{J_{i,1}}} \ \ t_{i,j}\le z_{i} \\ & &\forall_{1\le i\le p} \ \ \forall_{j\in J_{i,0}\cup J_{i,1}\cup\overline{J_{i,0}}\cup\overline{J_{i,1}}} \ \ t_{i,j}\le x_{j} \\ & &\forall_{1\le i\le p} \ \ \forall_{j\in J_{i,0}\cup J_{i,1}\cup\overline{J_{i,0}}\cup\overline{J_{i,1}}} \ \ t_{i,j}\ge x_{j} + z_{i} -1 \\ & &\forall_{1\le i\le p} \ \ \forall_{j\in \overline{J_{i,0}}\cup\overline{J_{i,1}}} \ \ u_{i,j}\le z_{i} \\ & &\forall_{1\le i\le p} \ \ \forall_{j\in \overline{J_{i,0}}\cup\overline{J_{i,1}}} \ \ u_{i,j}\le y_{j} \\ & &\forall_{1\le i\le p} \ \ \forall_{j\in \overline{J_{i,0}}\cup\overline{J_{i,1}}} \ \ u_{i,j}\ge y_{j} + z_{i} -1 \\ & & \text{all the variables \(x_{j}\), \(y_{j}\), \(z_{i}\), \(t_{i,j}\), \(u_{i,j}\)~:~binary} \end{array} $$

Let *I* be the set of all *j*∈{1,…,*q*} such that the *j*-th column of $\tilde {B}$ is known to be intrinsically heterozygous. The number of variables in NewModelG is $p + 2q - |I| + \sum _{i=1}^{p}\left (|J_{i,0}|+|J_{i,1}| + 2|\overline {J_{i,0}}| + 2|\overline {J_{i,1}}|\right)$ and so is that in OldModelG. Moreover, the number of constraints in NewModelG is $\sum _{i=1}^{p} \left (|J_{i,0}| + |J_{i,1}| + 2|\overline {J_{i,0}}| + 2|\overline {J_{i,1}}|\right)$, while that in OldModelG is $\sum _{i=1}^{p} \left (3|J_{i,0}| + 3|J_{i,1}| + 6|\overline {J_{i,0}}| + 6|\overline {J_{i,1}}|\right)$. So, NewModelG has significantly fewer constraints. Thus, it looks very promising that NewModelG can almost always be solved in much shorter time. In a later section, we will see that this is actually the case.

## Results and discussion

To evaluate the efficiency of our new models and compare them against the previous bests, we have implemented both our models and Chen et al.’s models [27] with CPLEX-v12.6.3 (an ILP solver by IBM). In our experiments, we use both real datasets and simulated datasets among which some include hard instances, because we expect that the new and the old models show significant difference in performance for hard instances. Since solving hard instances takes long time and we want to speed up our experiments, we use two Linux (x64) desktop PCs in our experiments. One PC has Intel Xeon E5-2687W v4 CPU (3.00 GHz, 48 threads) and 252 GB RAM, while the other has Intel i7-3960X CPU (3.30 GHz, 12 threads) and 32 GB RAM. The former is used to solve real datasets, while the latter is used to solve simulated datasets. To guarantee that we can finish the experiments within a reasonable amount of time, we put a time limit of 1 day on the running time of CPLEX for each reduced block.

CPLEX is always fast enough to find a solution within the time limit, albeit it is not necessarily optimal. However, in general, there is no guarantee on the MEC score of the solution when CPLEX fails to optimally solve within the time limit. Nonetheless, for the datasets used in our experiments, we will explicitly state (in tables) the (total) MEC scores of solutions found by CPLEX even when CPLEX fails to optimally solve one or more reduced blocks within the time limit. As will be seen from the tables, the MEC scores are quite close to the optimal when CPLEX fails.

### Results for real datasets

The real datasets used in our experiments are HuRef [17] and MP1 [28]. Both of them have been extensively used in previous studies to compare previous methods against each other [14, 17, 19–21, 27].

#### The HuRef dataset

This dataset is known to contain very hard instances. Indeed, He et al.’s program [19] fails to completely solve the all-heterozygous case of the problem for HuRef after taking 15 h on a PC cluster. Chen et al.’s program [27] based on ILP was the first to completely solve the all-heterozygous case of the problem for HuRef on a desktop PC. It turns out that our new models lead to much shorter time than Chen et al.’s models [27]. More specifically, Table 5 compares the performance of CPLEX on our new ILP models against that on Chen et al.’s old ILP models [27].

**Table 5:** Comparing the performance of CPLEX on our new ILP models against that on Chen et al.’s models [27] for HuRef

All-heterozygous Case			General Case			
Opt	Old	New	Old		New	
MEC	Time	Time	MEC	Time	MEC	Time
19665	7301	818	16853	80665	16853	7280
14647	1418	218	12618	1532	12618	374
10688	1221	156	9296	1236	9296	276
11537	1421	202	9958	1481	9958	351
10558	1550	211	9195	1770	9195	358
9884	1344	150	8637	1389	8637	270
11246	1362	182	9782	1504	9782	345
9800	2104	280	8480	3042	8480	848
9264	882	107	8051	956	8051	229
9815	1568	237	8550	1772	8550	431
8179	1156	169	7027	1193	7027	269
8213	921	135	7136	891	7136	235
5811	741	114	5090	788	5090	163
5844	545	70	5086	571	5086	145
9310	**87425**	11006	**8130**	**89135**	**8093**	**87551**
8235	1487	209	7176	1883	7176	445
6535	681	96	5739	756	5739	159
5019	789	94	4403	946	4403	168
5311	606	96	4628	629	4628	155
3741	371	55	3243	453	3243	93
3896	658	105	3360	1081	3360	370
4507	24327	592	**3915**	**86746**	3908	39866

Note: The *i*-th row shows the result for the *i*-th chromosome in HuRef, Old (respectively, New) means that the result is obtained with the old (respectively, new) model, MEC means the MEC score, MEC scores in bold mean non-optimal MEC scores, Time means the running time (in seconds) taken by CPLEX, and running time in bold means that CPLEX failed to optimally solve at least one reduced block of the chromosome within the time limit

We first focus on the all-heterozygous case. As can be seen from Table 5, CPLEX can solve NewModelA **9 times faster** than OldModelA on average. Indeed, CPLEX failed to optimally solve OldModelA for one reduced block of Chromosome 15 within the time limit. If we exclude Chromosome 15, then CPLEX can solve NewModelA **12 times faster** on average. In particular, for Chromosome 22, CPLEX can solve NewModelA **41 times faster**.

We next focus on the general case. As can be seen from Table 5, CPLEX can solve NewModelG **twice faster** on average. Indeed, CPLEX failed to optimally solve NewModelG for one reduced block of Chromosome 15 within the time limit, while CPLEX failed to optimally solve OldModelG for a total of two reduced blocks of Chromosomes 15 and 22 within the time limit. If we exclude the two chromosomes, then CPLEX can solve NewModelG **8 times faster** on average. In particular, for Chromosome 1, CPLEX can solve NewModelG **11 times faster**.

#### The MP1 dataset

MP1 is the most complete haplotype-resolved genome generated by fosmid pool-based next generation sequencing. The completeness of its phasing allows the determination of the molecular haplotype pairs for 81% of all autosomal protein-coding genes and provides the haploid DNA segments significantly larger than other standard shotgun sequencing technologies [34]. Solving the instances in MP1 optimally is much more difficult than solving those in HuRef optimally, because a number of chromosomes in MP1 contain very large reduced blocks with very large MEC scores. Previously, only Chen et al. [14] tried to optimally solve the all-heterozygous case for MP1. To the best of our knowledge, nobody has tried to optimally solve the general case for MP1.

As in [14], we focus on the all-heterozygous case for MP1. Chen et al. [14] assume that all columns of the input matrices in MP1 are heterozygous even if some of them are monotone. In contrary, we assume that monotone columns are homozygous and hence remove them from the input matrices when applying reductions to them. The removal of monotone columns makes the problem smaller and allow us to finish the experiments faster. In other words, the difference is only technical rather than essential. So, the MEC scores obtained in this paper will be smaller than those obtained in [14].

With NewModelA, we have tried to use CPLEX to optimally solve the all-heterozygous case for MP1. It turns out that NewModelA leads to much shorter time than OldModelA. More specifically, Table 6 compares the performance of CPLEX on NewModelA against that on OldModelA.

**Table 6:** Comparing the performance of CPLEX on NewModelA against that on OldModelA for MP1

Old			New		
MEC	Time	#Failed	MEC	Time	#Failed
110459	399777	3	110178	228176	0
105275	213509	2	104358	178884	2
96354	216205	1	93994	130262	1
94458	212936	2	92127	115174	1
75125	180227	1	75125	93795	0
89263	127016	1	87150	91412	1
78726	265535	2	78507	107233	0
63725	49251	0	63725	8342	0
55411	115714	1	55377	40680	0
67710	184078	1	67710	57863	0
68493	127354	1	67171	94397	1
56142	108465	1	55899	83974	0
39754	16250	0	39754	2130	0
39160	26624	0	39160	4197	0
41608	114950	1	41329	91593	1
58609	311474	1	58502	161319	1
43165	198344	1	43523	105746	1
33462	14725	0	33462	1944	0
33254	129098	1	32989	130484	1
35699	184073	1	35536	105899	1
34842	93111	1	34504	87497	1
16891	14775	0	16891	2067	0

Note: The *i*-th row shows the result for the *i*-th chromosome in MP1, #Failed means the number of reduced blocks not optimally solved by CPLEX within the time limit, and each other column means the same as in Table 5

As can be seen from Table 6, CPLEX can solve NewModelA almost **twice faster** than OldModelA on average. Indeed, CPLEX failed to optimally solve NewModelA for a total of 12 reduced blocks of 11 chromosomes within the time limit, while CPLEX failed to optimally solve OldModelA for a total of 22 reduced blocks of 17 chromosomes within the time limit. If we exclude the 17 chromosomes for which CPLEX failed to optimally solve OldModelA, then CPLEX can solve NewModelA **6.5 times faster** on average.

We note that the solutions found by CPLEX with OldModelA for the 5th and the 10th chromosomes happen to be optimal although CPLEX fails to finish within the time limit for some blocks of the two chromosomes; their optimality is witnessed by the results found by CPLEX with NewModelA. So, we suspect that with OldModelA, it takes too much time for CPLEX to verify the optimality of the solutions of some blocks of the two chromosomes.

### Simulated datasets

The simulated datasets used in our experiments are Geraci’s dataset [29] and Chen et al.’s Sim95_MP1 [14].

#### Geraci’s dataset

The dataset was generated from 3 parameters: the haplotype length *ℓ*∈{100,350,700}, the coverage *c*∈{3,5,8,10}, and the error rate *e*∈{0,0.1,0.2,0.3}. For each triple (*ℓ*,*c,e*), 100 matrices were generated. Intuitively speaking, the larger *c* (respectively, *e*) is, the more reads we have in the matrix (respectively, the larger optimal MEC score we have for the matrix).

As Chen et al. [27] did, we only use the matrices generated from (*ℓ,c,e*) with *ℓ*∈{100,350}, *c*∈{3,5,8,10}, and *e*=0.1, because these matrices contain both easy and difficult instances. It turns out that our new models lead to much shorter time than Chen et al.’s models [27]. More specifically, Table 7 compares the performance of CPLEX on our new ILP models against that on Chen et al.’s old ILP models [27].

**Table 7:** Comparing the performance of CPLEX on our new ILP models against that on Chen et al.’s models [27] for Geraci’s dataset

	Old		New	
(*ℓ*,*c*)	MEC	Time	MEC	Time
General Case				
(100,3)	66.58	1.31	66.58	0.06
(100,5)	132.9	14.42	132.9	3.4
(100,8)	249.39	78.95	249.39	19.61
(100,10)	327.23	177.98	327.23	39.16
(350,3)	233.46	46.69	233.46	10.35
(350,5)	477.2	409.91	477.2	97.3
(350,8)	878.63	3415.17	878.63	599.42
(350,10)	1152.31	16875.2	1152.31	1674.45
All-heterozygous Case				
(100,3)	53.31	0.23	53.31	0.03
(100,5)	95.38	7.62	95.38	3.4
(100,8)	157.34	30.82	157.34	17.75
(100,10)	195.83	52.54	195.83	26.51
(350,3)	186.22	35.67	186.22	15.24
(350,5)	338.4	216.23	338.4	74.12
(350,8)	547.06	817.12	547.06	337.4
(350,10)	682.09	1465.04	682.09	777.08

Note: MEC means the average MEC score (over 100 matrices), Time means the average running time of CPLEX (over 100 matrices), and Old and New mean the same as in Table 6

We first focus on the general case. As can be seen from Table 7, CPLEX can solve NewModelG **10 times faster** than OldModelG on average for hard instances (namely, those generated with (*ℓ*,*c*)=(350,10)). Indeed, CPLEX failed to optimally solve OldModelG for a total number of 2 reduced blocks of two instances generated with (*ℓ*,*c*)=(350,10) within the time limit. Even for the other (easier) instances, CPLEX can solve NewModelG at least **4 times faster** than OldModelG on average. We note that the solutions found by CPLEX with OldModelG for the two failed reduced blocks happen to be optimal, as witnessed by the results found by CPLEX with NewModelG. So, we suspect that with OldModelG, it takes too much time for CPLEX to verify the optimality of the solutions.

For the all-heterozygous case, the results in Table 7 show that CPLEX can solve our new model about **twice faster** on average.

#### Chen et al.’s Sim95_MP1

The matrices in MP1 are hard to solve to optimality. The main reason for this seems to be that the optimal MEC scores for the matrices are too large (i.e., the quality of the reads is bad). Since we believe that reads will be of higher quality in the future, we want to generate a simulated dataset from MP1 so that the reads cover the same SNP sites as in MP1 but have better quality. More specifically, Chen et al. [14] generated Sim95_MP1 from MP1 as follows. Initially, Sim95_MP1 is a copy of MP1. Then, we randomly partition the reads of Sim95_MP1 into two sets *R* and $\bar {R}$. For each read *r*∈*R*, we change each 1 in *r* to 0. On the other hand, for each read $r \in \bar {R}$, we change each 0 to 1. Now, the true solution for each matrix is simply a pair of complementary strings one of which consists of 0’s only. Finally, for each read *r* in *R* (respectively, $\bar {R}$) and each 0 (respectively, 1) of *r*, we flip the bit with a probability 1−*t*, where *t* is chosen by first generating it in normal distribution with mean 0.95 and standard deviation 0.05 and further resetting *t*=1 (respectively, *t*=0.6) if *t*>1 (respectively, *t*<0.6). Intuitively speaking, *t* is the quality of the bit and hence we want it to be around 0.95 and not smaller than 0.6.

With NewModelA, we have tried to use CPLEX to optimally solve the all-heterozygous case for Sim95_MP1. It turns out that NewModelA leads to much shorter time than OldModelA. More specifically, Table 8 compares the performance of CPLEX on NewModelA against that on OldModelA.

**Table 8:** Comparing the performance of CPLEX on NewModelA against that on OldModelA for Sim95_MP1

Old			New	
MEC	Time	#Failed	MEC	Time
84119	31622	0	84119	4003
88177	42667	0	88177	5000
75408	33375	0	75408	4114
77554	100474	1	74857	16470
65056	20386	0	65056	2783
70831	110238	1	70810	14465
64202	36747	0	64202	3481
56851	19366	0	56851	2397
47127	11598	0	47127	1745
56282	20768	0	56282	2728
56466	57839	0	56466	5229
49580	21330	0	49580	2423
35297	6258	0	35297	1044
35178	8522	0	35178	1233
33842	8136	0	33842	1173
40819	70744	0	40819	5019
30520	18319	0	30520	1862
30259	5973	0	30259	885
23422	31578	0	23422	2660
27374	13029	0	27374	1686
22920	89096	1	19205	10932
15014	3748	0	15014	540

Note: The *i*-th row shows the result for the *i*-th chromosome in Simu95_MP1 and each other column means the same as in Table 6

As can be seen from Table 8, CPLEX can solve NewModelA **8.3 times faster** than OldModelA on average. Indeed, CPLEX never failed to optimally solve NewModelA within the time limit, while CPLEX failed to optimally solve OldModelA for a total of 3 reduced blocks within the time limit.

## Conclusion

As aforementioned, solving the haplotype assembly problem to optimality is of great interest but very time-consuming. In order to speed up the computation of optimal solutions, we have designed better ILP models for both the all-heterozygous and the general cases of the problem. Our experimental results for both real and simulated datasets confirm that the new models can be solved within significantly shorter time than the previous bests. In this paper, we focused on finding optimal solutions. As future work, we plan to find out how much speed-up we can obtain when applying the models to speeding up heuristic algorithms.

## Abbreviations

FPT: Fixed-parameter tractable
ILP: Integer Linear Programming
LHR: Longest Haplotype Reconstruction
MaxSAT: the Maximum Satisfiability problem
MEC: Minimum Error Correction
MFC: Maximum Fragments Cut
MFR: Minimum Fragment Removal
MIFR: Minimum Implicit Fragment Removal
MISR: Minimum Implicit SNP Removal
MSR: Minimum SNP Removal
SNP: Single Nucleotide Polymorphism
